# Transcriptomic analysis reveals the lipid metabolism-related gene regulatory characteristics and potential therapeutic agents for myocardial ischemia-reperfusion injury

**DOI:** 10.3389/fcvm.2024.1281429

**Published:** 2024-01-29

**Authors:** Jiahe Wu, Huanhuan Cai, Xiaorong Hu, Wei Wu

**Affiliations:** ^1^ Department of Cardiology, Zhongnan Hospital of Wuhan University, Wuhan, China; ^2^ Institute of Myocardial Injury and Repair, Wuhan University, Wuhan, China; ^3^ Department of Clinical Laboratory, Institute of Translational Medicine, Renmin Hospital of Wuhan University, Wuhan, China

**Keywords:** myocardial ischemia-reperfusion injury, lipid metabolism, lipid metabolism-related genes, therapeutic agent, bioinformatics analysis

## Abstract

**Background:**

Impaired energy balance caused by lipid metabolism dysregulation is an essential mechanism of myocardial ischemia-reperfusion injury (MI/RI). This study aims to explore the lipid metabolism-related gene (LMRG) expression patterns in MI/RI and to find potential therapeutic agents.

**Methods:**

Differential expression analysis was performed to screen the differentially expressed genes (DEGs) and LMRGs in the MI/RI-related dataset GSE61592. Enrichment and protein-protein interaction (PPI) analyses were performed to identify the key signaling pathways and genes. The expression trends of key LMRGs were validated by external datasets GSE160516 and GSE4105. The corresponding online databases predicted miRNAs, transcription factors (TFs), and potential therapeutic agents targeting key LMRGs. Finally, the identified LMRGs were confirmed in the H9C2 cell hypoxia-reoxygenation (H/R) model and the mouse MI/RI model.

**Results:**

Enrichment analysis suggested that the “lipid metabolic process” was one of the critical pathways in MI/RI. Further differential expression analysis and PPI analysis identified 120 differentially expressed LMRGs and 15 key LMRGs. 126 miRNAs, 55 TFs, and 51 therapeutic agents were identified targeting these key LMRGs. Lastly, the expression trends of Acadm, Acadvl, and Suclg1 were confirmed by the external datasets, the H/R model and the MI/RI model.

**Conclusion:**

Acadm, Acadvl, and Suclg1 may be the key genes involved in the MI/RI-related lipid metabolism dysregulation; and acting upon these factors may serve as a potential therapeutic strategy.

## Introduction

Early successful reperfusion therapy is the key measure to reduce the size of myocardial infarct area and rescue dying myocardial cells (1). However, restoration of blood perfusion after sustained myocardial ischemia can further exacerbate structural and functional myocardial damages, leading to myocardial stunning, reduced cardiac function, and malignant arrhythmias. This is generally known as myocardial ischemia-reperfusion injury (MI/RI) (2, 3). It has been reported that the damaged area caused by MI/RI would account for up to 50% of size from the overall damaged area (4). The severity of MI/RI can be affected by many factors, such as the size of the ischemic area, the duration of ischemia, reperfusion flow rate, and oxygen content (5). The mechanism of MI/RI is very complex and involves oxidative stress, inflammatory response, energy metabolism disorder, mitochondrial damage, Ca^2+^ overload, apoptosis, and other biological processes (6–8). It is important to note that there are currently no clinically effective prevention or treatment measures for MI/RI.

Lipid metabolism process including the synthesis, storage, breakdown, and utilization of lipids, plays an important role in maintaining cellular homeostasis and energy balance (5). Under physiological conditions, 50%–70% of the heart's energy support comes from the fatty acid β-oxidation process (9). In the setting of myocardial ischemia, the uptake, and utilization of fatty acids in cardiomyocytes are increased to maintain the energetic supply; while the decreased oxygen supply leads to the inhibition of fatty acid β-oxidation and ultimately increases the level of fatty acid intermediates metabolites. When reperfusion occurs, the increased fatty acid β-oxidation causes increased oxidative stress triggering cell death and MI/RI exacerbations (10).

Dysregulation in lipid metabolism is usually characterized by abnormalities in several associated enzymes, transporters, and regulatory proteins. Lipid transporter CD36 is a key regulator mediating cellular uptake of long-chain fatty acids. CD36 knockdown in the heart can significantly inhibit fatty acid uptake and oxidation, and reduce MI/RI (11). Malonyl CoA can also inhibit mitochondrial fatty acid uptake, effectively reduce acidosis, thus, impedes MI/RI (12). ALOX15 promotes the reaction of polyunsaturated fatty acids-phospholipids peroxidation, increases susceptibility to ferroptosis, and promotes MI/RI (13). Cardiomyocyte-specific knockout calcium-independent phospholipase A2γ can significantly reduce fatty acid oxidation and infarct size in MI/RI (14). These studies suggested that lipid metabolism-related genes (LMRGs) could play an important role in MI/RI, and an effective intervention based on these genes may be a potential strategy to prevent or treat MI/RI. Therefore, it is necessary to explore the mode of action of LMRGs in MI/RI and find potential therapeutic targets.

**Figure 1:**
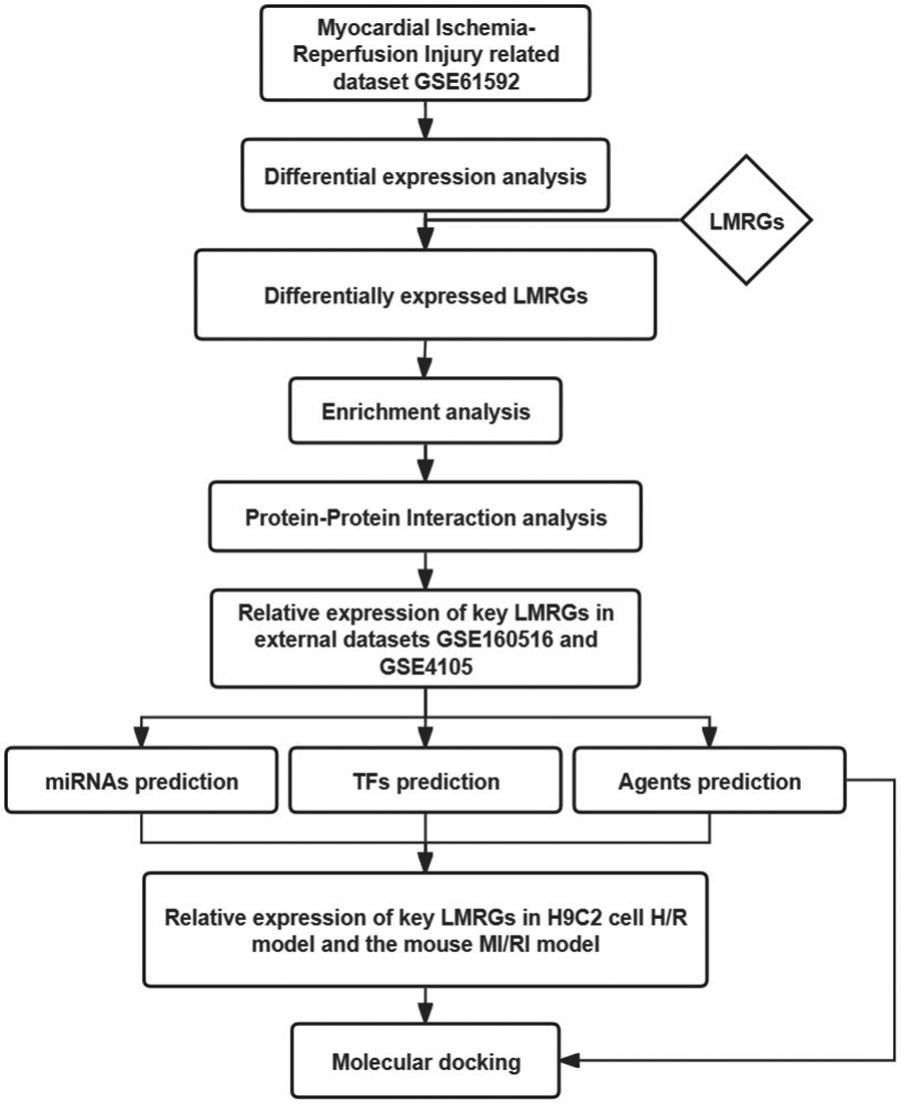
Flowchart of the steps performed in this study.

This study used bioinformatics methods to screen the LMRGs in MI/RI-related datasets. The mechanism of these genes was explored by enrichment analysis. The miRNAs, transcription factors (TFs), and therapeutic agents targeting key LMRGs were predicted by relevant databases. This study may provide new clues for further exploring the mechanism of lipid metabolism involved in MI/RI, aiming to unveil the attached biological process and discover new potential therapeutic agents. Figure 1 shows the flowchart of the steps performed in this study.

## Methods

### Data acquisition and collation

The original gene expression profile data of MI/RI-related datasets GSE61592 (mouse), GSE160516 (mouse), and GSE4105 (rat) was obtained from the Gene Expression Omnibus (GEO) database (https://www.ncbi.nlm.nih.gov/). These datasets contained the following characteristics: (1) the species was Mus musculus or Rattus norvegicus; (2) The experimental group underwent MI/RI treatment; (3) The samples were obtained from myocardial tissue; (4) Complete raw gene expression profiling data were provided. The GSE61592 originated from the GPL6887 platform Illumina MouseWG-6 v2.0 expression beadchip. GSE160516 originated from the GPL23038 platform [Clariom_S_Mouse] Affymetrix Clariom S Assay, Mouse (Includes Pico Assay). GSE4105 originated from the GPL341 platform [RAE230A] Affymetrix Rat Expression 230A Array (15). GSE61592 included 3 sham-operated mice and 3 MI/RI-model mice. In GSE61592, the left anterior descending coronary artery (LAD) was ligated for 90 min followed by 72 h of reperfusion to construct the MI/RI model; In GSE160516, the MI/RI model was established by 30 min of LAD ligation followed by reperfusion for 6 h, 24 h, and 72 h. The control group was sham-operated. Each group contained 4 mice; In GSE4105, rats underwent surgery for 30 min of LAD ligation followed by reperfusion for 2 days and 7 days. The control group was sham-operated. Each group contained 3 mice. In this study, GSE61592 was set as the analysis dataset, and GSE160516 and GSE4105 were set as the external validation datasets. Table 1 shows the details of the included datasets.

**Table 1:** The detailed information of the 3 microarray datasets.

Data source	Organism	Platform	Year	Sample source	Sample size (CON: MI/RI)	Detected RNA type
GSE61592	Mus musculus	GPL6887	2015	Myocardial tissue	3:3	mRNA
GSE160516	Mus musculus	GPL23038	2020	Myocardial tissue	4:12	mRNA
GSE4105	Rattus norvegicus	GPL341	2006	Myocardial tissue	6:6	mRNA

### Identification of differentially expressed genes and differentially expressed LMRGs in myocardial ischemia-reperfusion injury

To screen differentially expressed genes (DEGs) between MI/RI and Sham controls, the “limma package” of R software (version 4.0.1) was used to perform differential expression analysis on the MI/RI-related dataset GSE61592. The *P*-value was corrected by the Benjamini–Hochberg method and the screening threshold of DEGs was set as adj. *P*-value <0.05 and |logFC| ≥ 1.5. Previous studies on bioinformatics analysis of lipid metabolism were retrieved and integrated to obtain a list of LMRGs (16–19). Finally, 1,454 LMRGs were identified for subsequent analysis (Supplementary File S1). The online Venn diagram website (http://bioinformatics.psb.ugent.be/webtools/Venn/) intersected these LMRGs with DEGs to obtain the differentially expressed LMRGs.

### Go annotation and KEGG pathway enrichment analysis

Gene Ontology (GO) annotation and Kyoto Encyclopedia of Genes and Genomes (KEGG) pathway enrichment analysis were completed with the “cluster Profiler package” of R software (version 4.0.1) (20, 21). GO analysis includes three parts: biological process (BP) analysis, cellular component (CC) analysis, and molecular function (MF) analysis. The screening criterion for enrichment analysis results was *P* < 0.05.

### Construction of the protein-protein interaction network and identification of key genes

Protein-protein interaction (PPI) analysis was performed by String (https://cn.string-db.org) online database to explore the interaction between the proteins expressed by the identified LMRGs. The Cytoscape software (version 3.10.0) was used to visualize the PPI analysis results. Three algorithms (MCC, Degree, EPC) of the Cytohubba plugin were used to select and sort the top 20 genes respectively. Genes predicted by all three algorithms were identified as key LMRGs.

### Validation of key LMRGs in the myocardial ischemia-reperfusion injury related datasets GSE160516 and GSE4105

GSE160516 contains mouse myocardial expression profile data at 6 h, 24 h, and 72 h of reperfusion (*n* = 4 for each group). GSE4105 contains rat myocardial expression profile data at 2 days and 7 days of reperfusion (*n* = 3 for each group). Based on the original gene expression data in these two datasets, the expression trends of the identified key LMRGs were further verified. Independent sample *T*-test was used to compare the H/R and the control groups, and *P* < 0.05 was considered statistically significant.

### Prediction of key LMRGs-related miRNAs, transcription factors, and therapeutic agents

Five online databases Targetscan (version 7.2, https://www.targetscan.org/vert_72/), miRDB (https://mirdb.org/), miRWalk (http://mirwalk.umm.uni-heidelberg.de/), microT-CDS (https://dianalab.e-ce.uth.gr/html/dianauniverse/index.php?r=microT_CDS), and Tar Base (version 8, https://dianalab.e-ce.uth.gr/html/diana/web/index.php?r=tarbasev8) were used to predict miRNAs targeting key LMRGs (22, 23). miRNAs predicted by three or more databases at the same time were considered to target the gene. TFs that regulate the expression of key LMRGs were predicted by the Regnetwork database (https://regnetworkweb.org/). Finally, Comparative Toxico-genomics Database (CTD, https://ctdbase.org/) and CLUE (https://clue.io/) database (L1000 Platform) were used to predict therapeutic agents targeting key LMRGs (24, 25). CTD is a powerful publicly available database that provides manually curated information on chemo-gene/protein interactions, chemo-disease, and gene-disease relationships. CLUE contains the world's largest perturbation-driven gene expression dataset. Target agents included in literature or predicted by computer algorithms can be obtained by entering gene names in these two databases. Agents predicted by both databases were considered the targeted agents for the gene. Agents targeting more than four genes at the same time are considered potential Agents for the treatment of MI/RI.

### Cardiomyocyte cell line culture and hypoxia-reoxygenation treatment

Rat cardiomyocyte H9C2 cells were purchased from BeNa Culture Collection (https://www.bncc.com/bncc/plist/p1-1-38-1.html, BNCC337726, Beijing, China). The cells were cultured in DMEM medium (Gibco, Invitrogen, Carlsbad, CA, USA) with 10% fetal bovine serum (FBS, Gibco, Australia) and 1% penicillin-streptomycin (Sigma-Aldrich, St. Louis, MO, USA). After the cells had grown to a suitable density, they were transferred to the three-gas incubator for 12 h of hypoxia (1% O2, 5% CO2, and 94% N2; 37°C), followed by 4 h of reoxygenation. The cells in the control group were always cultured under normal conditions (21% O2, 5% CO2, and 74% N2; 37°C).

### Animal acquisition and myocardial ischemia-reperfusion treatment

All animal experiments met the standards of the Care and Use of Laboratory Animals of the National Institutes of Health. The experimental protocol was ethically reviewed and approved by the Animal Experiment Center of Wuhan University (Number: ZN2023149). The C57BL/6 wild-type mice (male, specific pathogen-free, 8 weeks old) were purchased from the Shanghai Animal Laboratory Center and randomly divided into sham operation group and MI/RI model group (*n* = 6 for each group). The surgical method was referred to previous literature (26–28), and the mouse model of MI/RI was established by ligating the LAD for 30 min followed by reperfusion (Remove the No. 10 polyethylene tube inside the ligating coil). Myocardial tissues were obtained 24 h after reperfusion for subsequent detection and validation.

### Evaluation of the mouse myocardial ischemia-reperfusion injury model

To further verify the success of mouse MI/RI model construction, we used 2, 3, 5-triphenyltetrazolium chloride (TTC) staining to detect myocardial infarct size, Hematoxylin-eosin (H&E) staining to detect myocardial morphology, terminal-deoxynucleotidyl transferase-mediated 2′-deoxyuridine 5′-triphosphate (dUTP) nick end labeling (TUNEL) staining to detect apoptosis levels, echocardiography to evaluate ejection function of the mouse heart, and Lactate dehydrogenase (LDH) kit to detect plasma LDH levels. The detailed model construction and validation methods are presented in Supplementary File S2.

### Real-time quantitative polymerase chain reaction

RT-qPCR was used to verify the expression trends of the identified key LMRGs in the hypoxia-reoxygenation (H/R) model and the mouse MI/RI model. According to the manufacturer's instructions, total RNA extraction, reverse transcription, and RT-qPCR reactions were performed using kits FastPure® Cell/Tissue Total RNA Isolation Kit V2 (Vazyme, Nanjing, China), Hifair® Ⅲ 1st Strand cDNA Synthesis SuperMix for qPCR (YEASEN, Shanghai, China), and Hieff UNICON® Universal Blue qPCR SYBR Green Master Mix (YEASEN, Shanghai, China), respectively. The RT-qPCR reaction process was carried out in the Bio-Rad Connect Real-time PCR Detection System. The expression differences of key LMRGs between the H/R group and the control group were compared with β-Actin as the reference gene (29). The expression differences of key LMRGs between the MI/RI group and the Sham group were compared with Gapdh as the reference gene (30). Relative change multiples were calculated using the 2^−*ΔΔ*Ct^ method. Details of the primers used for the reaction are shown in Supplementary File S3. Data were presented as mean values ± standard error of the mean (SEM) from at least three independent experiments. Independent sample *T*-test was used to compare the experimental and the control groups, and *P* < 0.05 was considered statistically significant.

### Western blotting

Frozen mouse myocardial tissues were homogenized using RIPA lysis buffer (Sigma–Aldrich, St. Louis, MI, United States) supplemented with PMSF (Beyotime, Shanghai, China) and Phosphatase Inhibitor Cocktail (CWBIO). Crude fractions were centrifuged at 12,000 × g for 15 min at 4°C, and then the supernatants were immediately collected. Aliquots of 30 μg of protein were separated by SDS-PAGE, transferred onto PVDF membranes (Millipore, Bedford, MA, United States), blocked with 5% non-fat milk for 1 h, and then incubated with primary antibodies at 4°C overnight. Antibody dilutions were 1:1,000 for Acadm (A4567, ABclonal, Wuhan, China), 1:2,000 for Acadvl (A7865, ABclonal, Wuhan, China) and Suclg1 (A15345, ABclonal, Wuhan, China), and 1:10,000 for Gapdh (AC002, ABclonal, Wuhan, China). The membranes were then incubated with horseradish peroxidase-conjugated secondary antibodies at room temperature for 1 h. Signals were detected with ECL (Tanon, Shanghai, China).

### Molecular docking

The 3D structures of the predicted agents were found in the PubChem (https://pubchem.ncbi.nlm.nih.gov/) database and saved as an SDF file. The 3D structures of proteins expressed by key LMRGs were searched in the RCSB PDB database (https://www.rcsb.org/) and downloaded in PDB format. These structural files of proteins and predicted agents were uploaded in CB-Dock2 (https://cadd.labshare.cn/cbdock2/php/blinddock.php#job_list_load) and subsequently performed for molecular docking (31). The molecular docking model was evaluated by calculating the Vina score, and a lower Vina score of the model indicated a more stable binding.

## Results

### Identification of differentially expressed genes and differentially expressed LMRGs in myocardial ischemia-reperfusion injury

**Figure 2:**
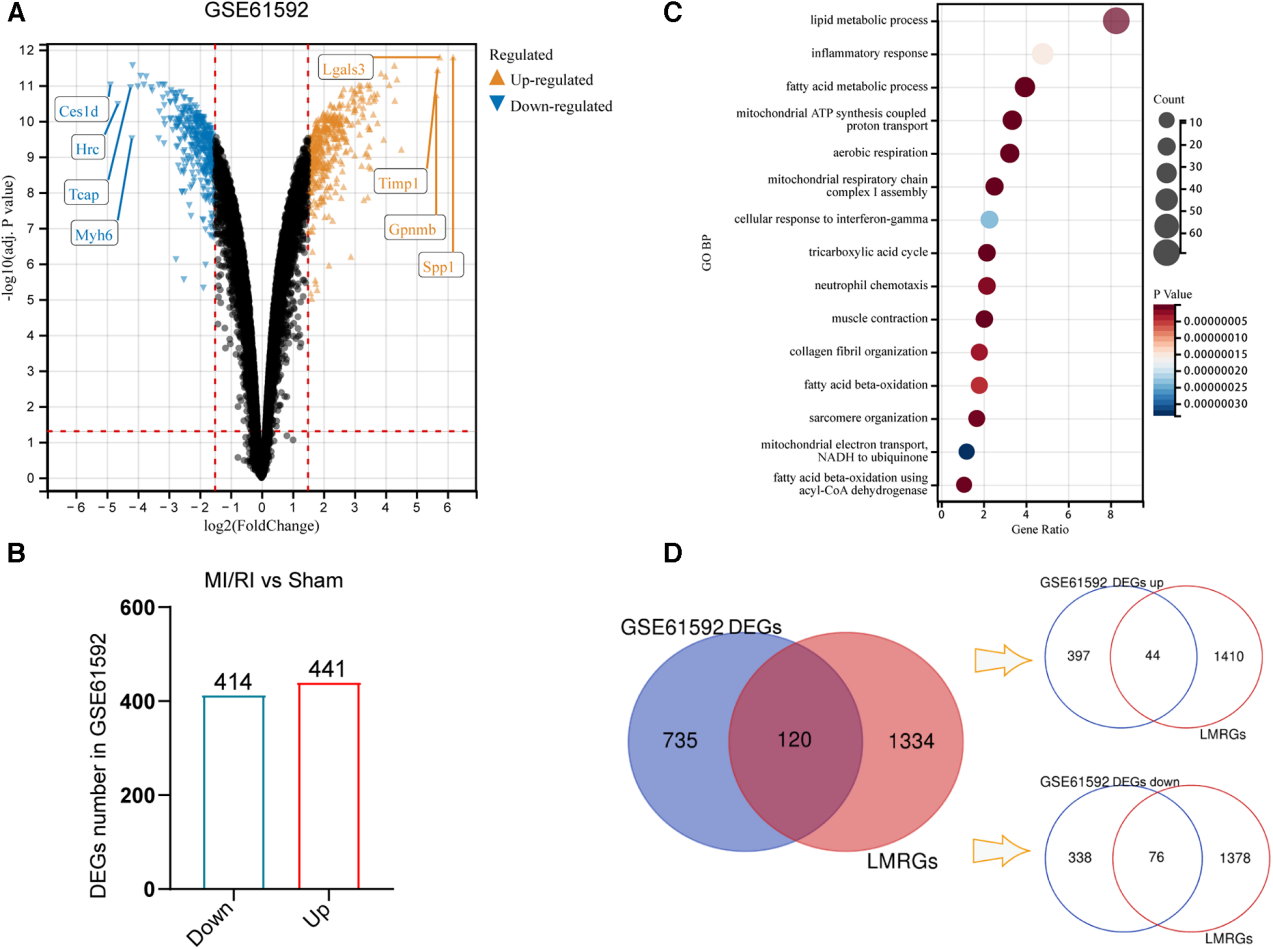
Identification of differentially expressed genes and differentially expressed LMRGs in myocardial ischemia-reperfusion injury. (**A**) The volcano plot of differential expression analysis for GSE61592. (**B**) DEGs number in GSE61592. (**C**) The Bubble plot of GO BP analysis results for DEGs in GSE61592. (**D**) Venn diagram for the identification of differentially expressed LMRGs.

Differential expression analysis was performed on the MI/RI-related dataset GSE61592 to compare the differences in gene expression between MI/RI and sham controls. With “adj. *P*-value <0.05 and |logFC| ≥ 1.5” as the threshold, 855 DEGs were identified, of which 414 were down-regulated, and 441 were up-regulated (Figures 2A,B). Figure 2A shows the volcano plot of the differential expression analysis. Supplementary File S4 shows detailed information on 855 DEGs. GO BP enrichment analysis suggested that lipid metabolism was a key biological process involved in MI/RI (Figure 2C). The genes in the LMRGs list (Supplementary File S1) were intersected with the DEGs to obtain the differentially expressed LMRGs. Finally, 120 differentially expressed LMRGs were identified, of which 44 were up-regulated and 76 were down-regulated (Figure 2D).

### Go annotation and KEGG pathway enrichment analysis of LMRGs in myocardial ischemia-reperfusion injury

**Figure 3:**
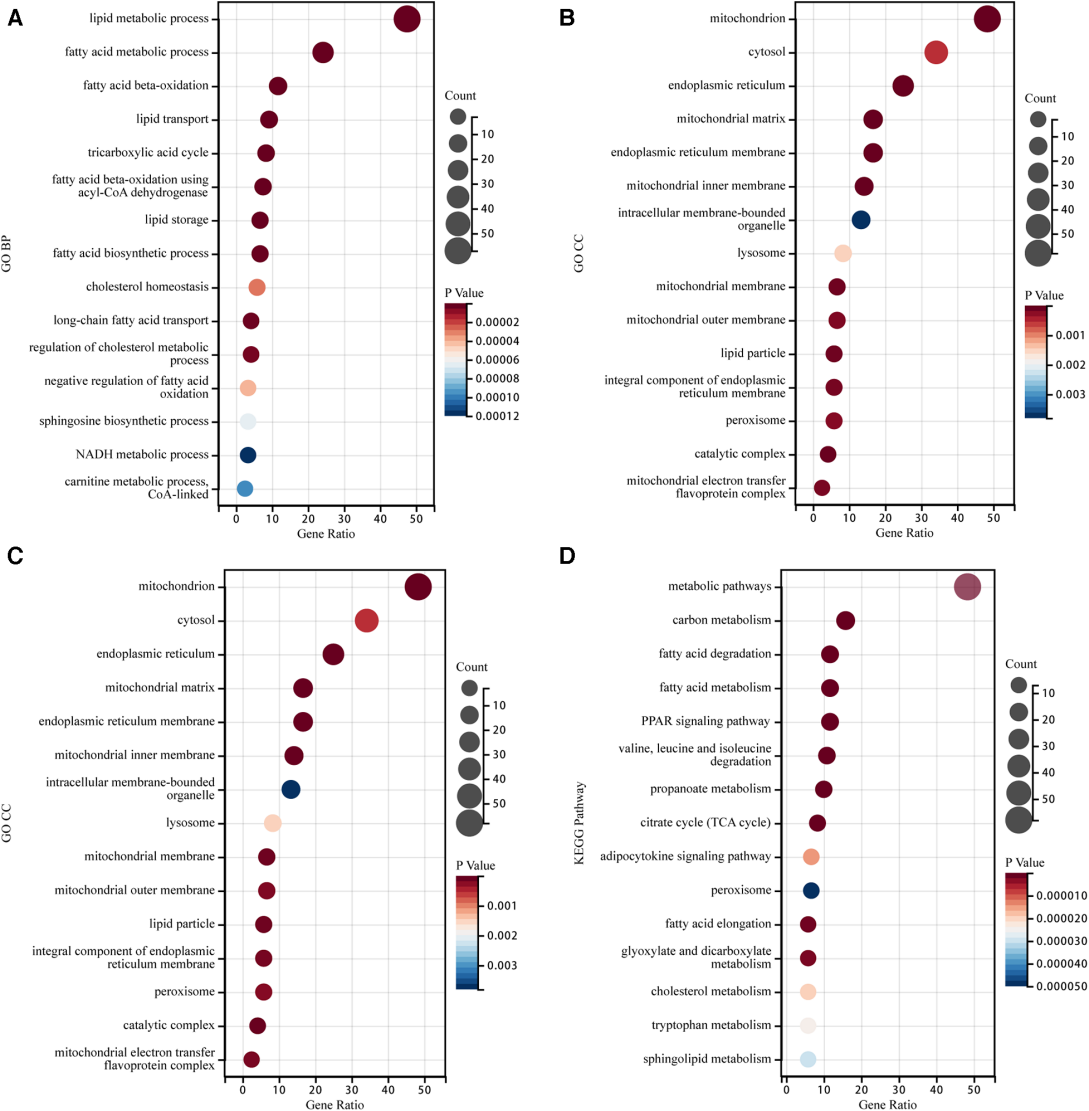
Go annotation and KEGG pathway enrichment analysis of LMRGs in myocardial ischemia-reperfusion injury. (**A**) The Bubble plot of GO BP analysis results for differentially expressed LMRGs. (**B**) The Bubble plot of GO CC analysis results for differentially expressed LMRGs. (**C**) The Bubble plot of GO MF analysis results for differentially expressed LMRGs. (**D**) The Bubble plot of KEGG analysis results for differentially expressed LMRGs.

Enrichment analysis was performed on the identified LMRGs to explore their biological processes and signaling pathways. BP analysis of GO annotation showed these LMRGs mainly involved in the terms of “lipid metabolic process,” “fatty acid beta-oxidation,” “lipid transport,” “lipid storage,” and “fatty acid biosynthetic process,” etc (Figure 3A). CC analysis of GO annotation illustrated that the proteins expressed by these LMRGs were mainly localized in “mitochondrion”, “cytosol”, “endoplasmic reticulum”, “mitochondrial matrix”, and “endoplasmic reticulum membrane”, etc (Figure 3B). GO MF analysis illustrated that these LMRGs would mainly carry out functions such as “oxidoreductase activity,” “transferase activity,” “ligase activity,” “isomerase activity,” “acyl-CoA dehydrogenase activity,” etc (Figure 3C). According to the KEGG pathway enrichment analysis, the identified LMRGs were mainly involved in signaling pathways such as “fatty acid degradation,” “PPAR signaling pathway,” “adipocytokine signaling pathway,” “peroxisome,” and “cholesterol metabolism,” etc (Figure 3D).

### Construction of the protein-protein interaction network and identification of key genes

**Figure 4:**
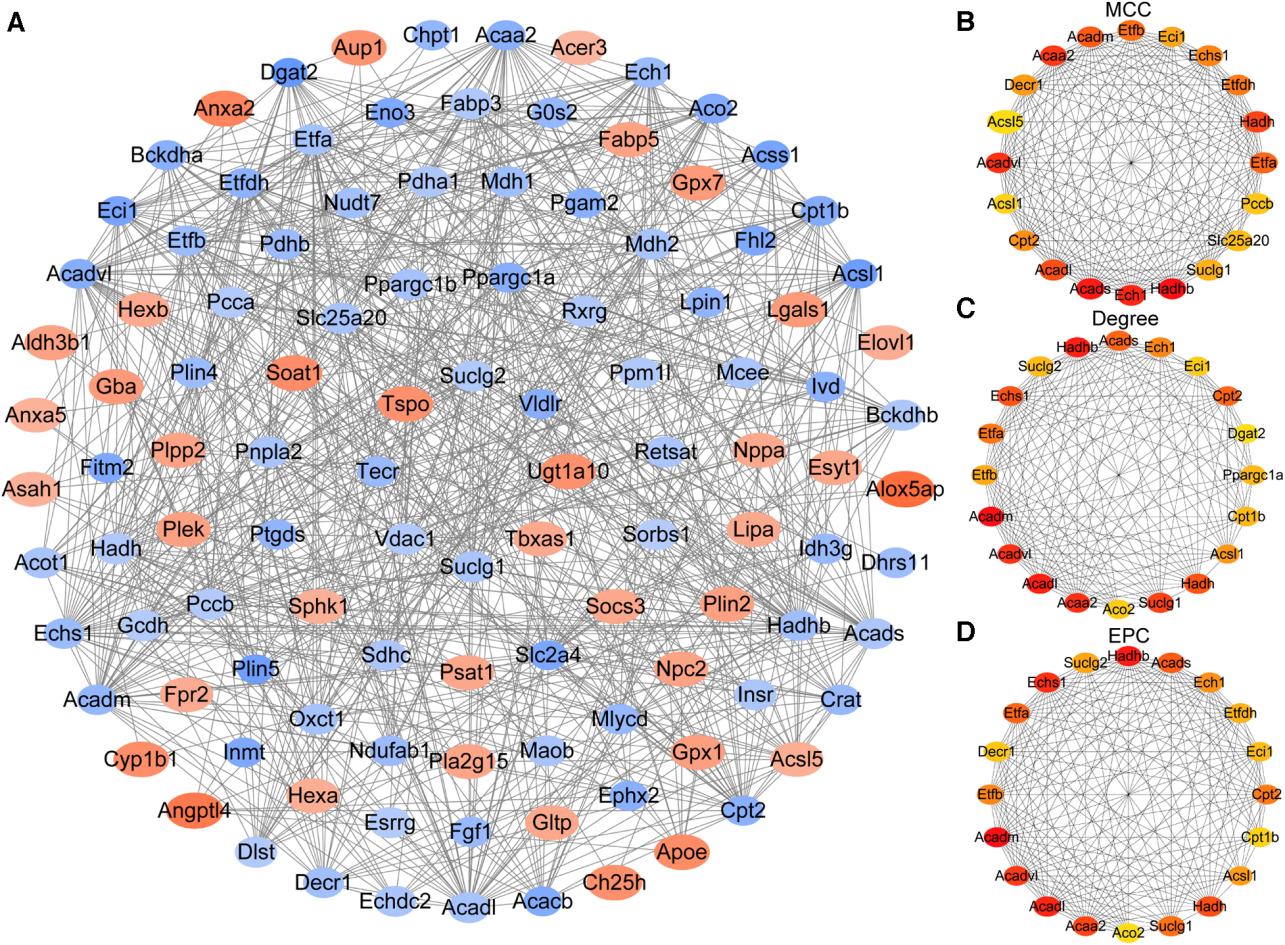
Construction of the protein-protein interaction network and identification of key genes. (**A**) The whole PPI Network of differentially expressed LMRGs. (**B**) Prediction results of the MCC algorithm. (**C**) Prediction results of the Degree algorithm. (**D**) Prediction results of the EPC algorithm.

PPI analysis was performed by the String database to explore the interaction between the proteins expressed by the identified LMRGs. As a result of the analysis, the Cytoscape software constructed a PPI network with 111 nodes and 744 edges (version 3.10.0) (Figure 4A). Three algorithms (MCC, Degree, EPC) were used to predict key LMRGs simultaneously (Figures 4B–D and Supplementary File S5). Finally, 15 LMRGs (*Acaa2, Acadl, Acadm, Acads, Acadvl, Acsl1, Cpt2, Ech1, Echs1, Eci1, Etfa, Etfb, Hadh, Hadhb, and Suclg1*) were ranked in the top 20 in the respective prediction results of the three algorithms, which were considered as the key LMRGs.

### Validation of key LMRGs in the mouse myocardial ischemia-reperfusion injury related dataset GSE160516

**Figure 5:**
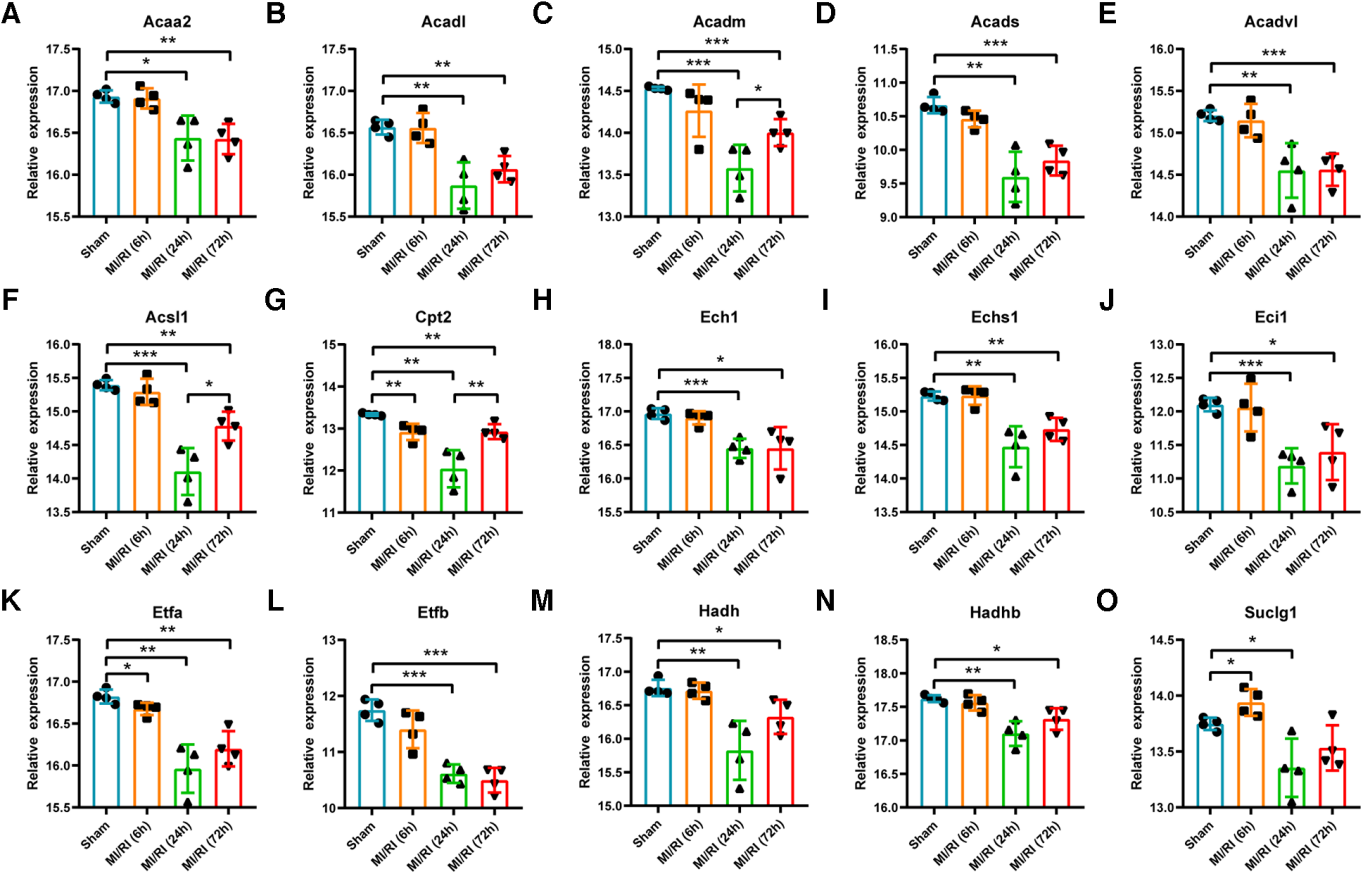
Validation of key LMRGs in the mouse myocardial ischemia-reperfusion injury related dataset GSE160516. (**A–O**) Validation of 15 key LMRGs Acaa2, Acadl, Acadm, Acads, Acadvl, Acsl1, Cpt2, Ech1, Echs1, Eci1, Etfa, Etfb, Hadh, Hadhb, and Suclg1 at 6 h, 24 h, and 72 h of reperfusion in GSE160516. i = 4 for each group. For panels (**A–O**) *P < 0.05, **P < 0.01, ***P < 0.001.

GSE160516 contained mouse myocardial expression profile data at 6 h, 24 h, and 72 h of reperfusion (*n* = 4 for each group). Based on this dataset's original gene expression profile data, the expression trends of key LMRGs were verified. The results showed that the expression levels of *Acaa2, Acadl, Acadm, Acads, Acadvl, Acsl1, Cpt2, Ech1, Echs1, Eci1, Etfa, Etfb, Hadh, and Hadhb* decreased significantly when the reperfusion time was 24 h and 72 h (Figures 5 A–N). Among them, *Cpt2* and *Etfa* decreased significantly at 6 h of reperfusion, and the expression of *Acadm, Acsl1, and Cpt2* partially recovered at 72 h compared with 24 h of reperfusion. In addition, the expression level of *Suclg1* increased significantly at 6 h of reperfusion and then decreased significantly at 24 h of reperfusion (Figure 5O).

### Validation of key LMRGs in the rat myocardial ischemia-reperfusion injury related dataset GSE4105

**Figure 6:**
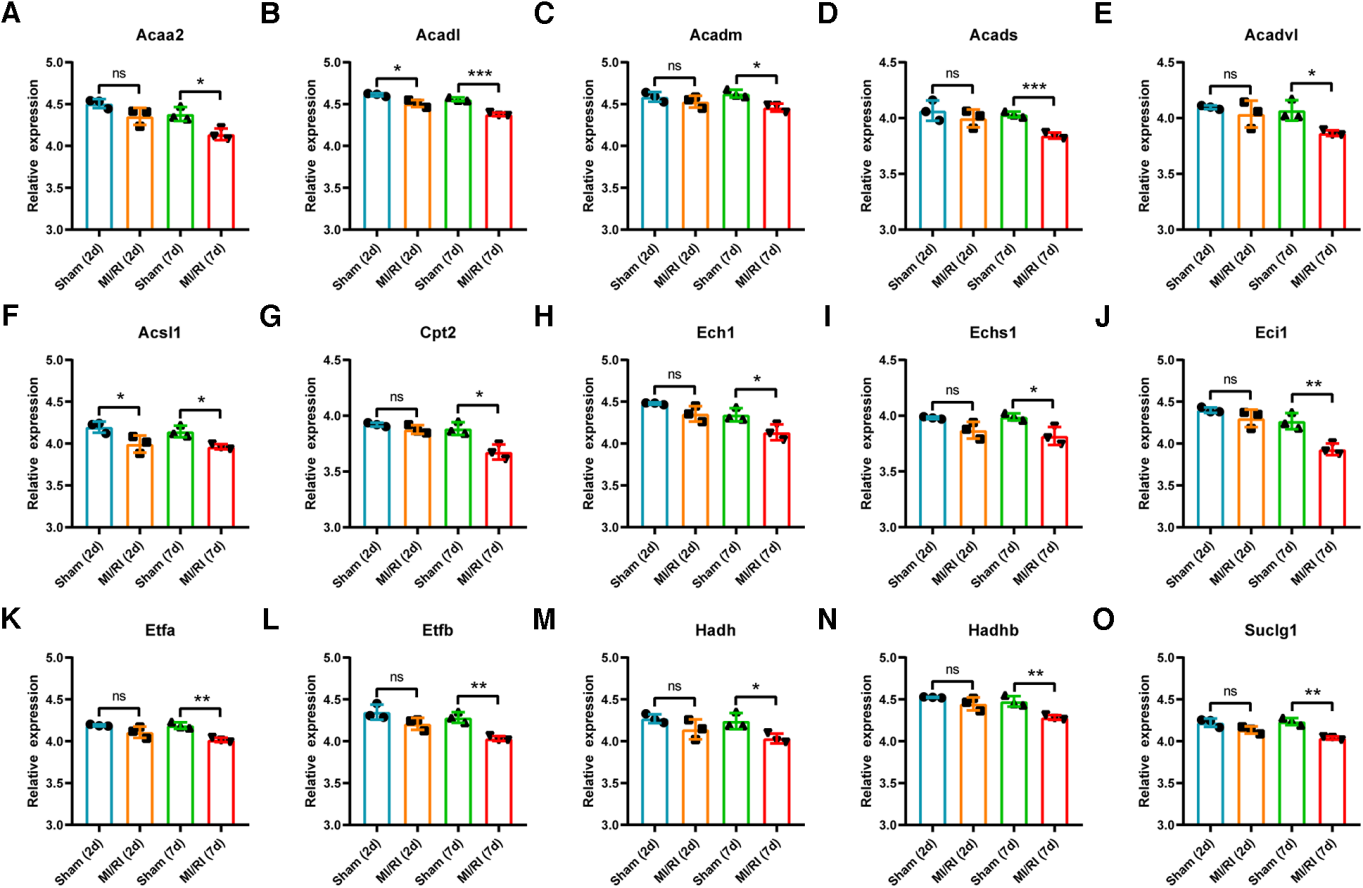
Validation of key LMRGs in the rat myocardial ischemia-reperfusion injury related dataset GSE4105. (**A–O**) Validation of 15 key LMRGs Acaa2, Acadl, Acadm, Acads, Acadvl, Acsl1, Cpt2, Ech1, Echs1, Eci1, Etfa, Etfb, Hadh, Hadhb, and Suclg1 at 2 days and 7 days of reperfusion in GSE4105. *n* = 3 for each group. For panels (**A–O**), ns indicates no statistical difference; * *P* < 0.05, **P < 0.01, ***P < 0.001.

GSE4105 contained rat myocardial expression profile data at 2 days and 7 days of reperfusion (*n* = 3 for each group). Based on this dataset's original gene expression profile data, the expression trends of key LMRGs were verified. As shown in Figures 6A–O, the expression of *Acaa2, Acadm, Acads, Acadvl, Acsl1, Cpt2, Ech1, Echs1, Eci1, Etfa, Etfb, Hadh, Hadhb, Suclg1* decreased significantly when the reperfusion time was 7 days. *Acadl and Acsl1* decreased significantly when the reperfusion time was 2 days and 7 days.

### Prediction of key LMRGs-related miRNAs, transcription factors, and therapeutic agents

**Figure 7:**
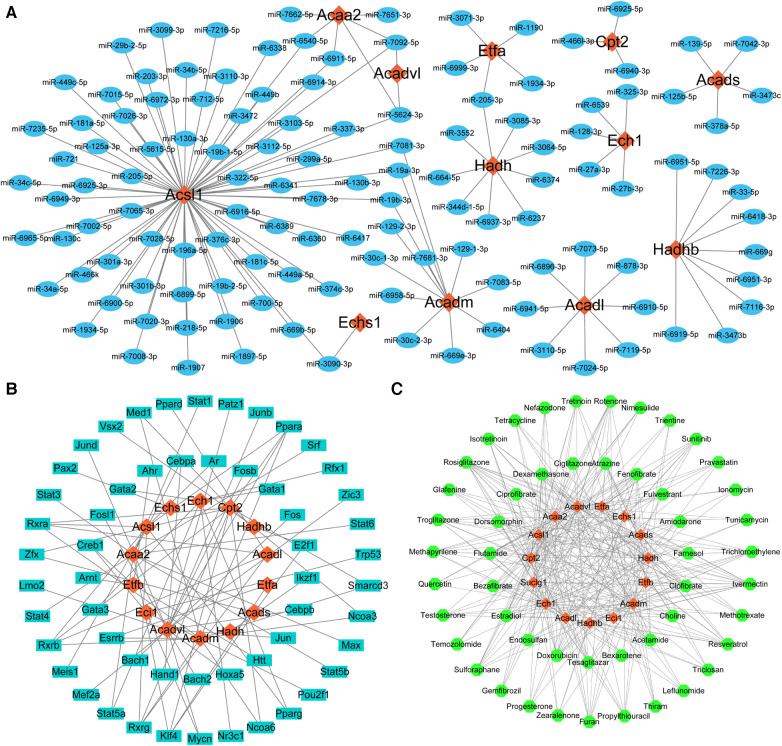
Prediction of key LMRGs-related miRNAs, transcription factors, and therapeutic agents. (**A**) The whole miRNA-mRNA regulatory network. (**B**) The whole TF-mRNA regulatory network. (**C**) The whole agent-mRNA regulatory network. For panels (**A–C**) The orange-red diamond nodes represent key LMRGs; the blue oval nodes represent miRNAs; the cyan oblong nodes represent TFs; the green circular nodes represent therapeutic agents.

MiRNAs, TFs, and therapeutic agents targeting the key LMRGs were predicted through the corresponding online databases. According to the predicted molecular regulatory relationships, the corresponding network diagrams were drawn using the Cytoscape software (version 3.10.0) (Figures 7A–C). MiRNA prediction identified 126 miRNAs that regulated these key LMRGs, and a miRNA-mRNA regulatory network containing 138 nodes and 137 edges was built (Figure 7A). Among the predicted miRNAs, miR-5624-3p, miR-7092-5p, miR-6540-5p, miR-6911-5p, miR-7081-3p, miR-19b-3p, miR-19a-3p, miR-3090-3p, miR-205-3p target two or more LMRGs simultaneously. TF prediction identified 55 TFs regulating these key LMRGs, and a TF-mRNA regulatory network containing 69 nodes and 82 edges was constructed (Figure 7B). Among the predicted TFs, *Ppara, Rxrb, Rxra, Rxrg, Klf4, Cebpa, Stat5a, Med1, Smarcd3, Ncoa3, Ncoa6, Esrrb, Zfx, E2f1, Mycn, Pparg, Creb1* target two or more LMRGs simultaneously. In addition, 51 potential therapeutic agents such as estradiol, fenofibrate, resveratrol, sulforaphane, sunitinib, bezafibrate, propylthiouracil, pravastatin, quercetin, ciprofibrate were predicted to target the identified key LMRGs and therefore may be potential agents for the treatment of MI/RI. Figure 7C is the agent-mRNA regulatory network showing the corresponding molecular regulatory relationship (65 nodes and 349 edges).

### Validation of the identified key LMRGs in H9C2 cell hypoxia-reoxygenation model

**Figure 8:**
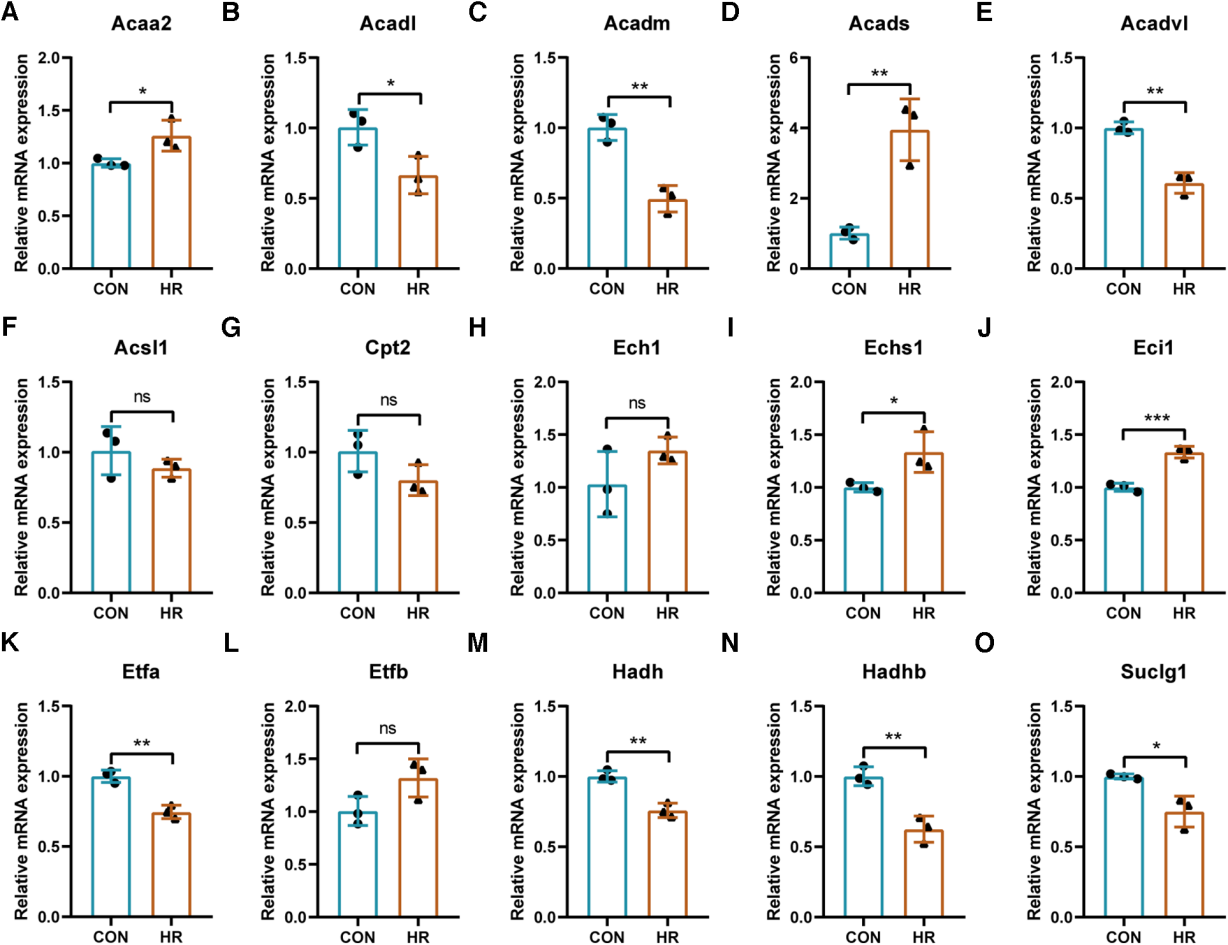
Validation of the identified key LMRGs in H9C2 cell hypoxia-reoxygenation model. (**A–O**) Validation of 15 key LMRGs Acaa2, Acadl, Acadm, Acads, Acadvl, Acsl1, Cpt2, Ech1, Echs1, Eci1, Etfa, Etfb, Hadh, Hadhb, and Suclg1 in the H9C2 cell H/R model. *n* = 3 for each group. For panels (**A–O**), ns indicates no statistical difference; *P < 0.05, **P < 0.01, ***P < 0.001.

The expression trends of the identified key LMRGs were verified in the H9C2 cell H/R model (Figures 8A–O). The results showed that 11 LMRGs were differentially expressed in the H/R model, among which *Acaa2, Acads, Echs1, and Eci1* were significantly up-regulated, while *Acadl, Acadm, Acadvl, Etfa, Hadh, Hadhb, and Suclg1* were significantly down-regulated. The expression trends of 7 down-regulated genes (*Acadl, Acadm, Acadvl, Etfa, Hadh, Hadhb, and Suclg1*) were consistent with the prediction results; while 4 up-regulated genes (*Acaa2, Acads, Echs1, and Eci1*) showed the opposite trends.

### Establishment and evaluation of the mouse myocardial ischemia-reperfusion injury model

**Figure 9:**
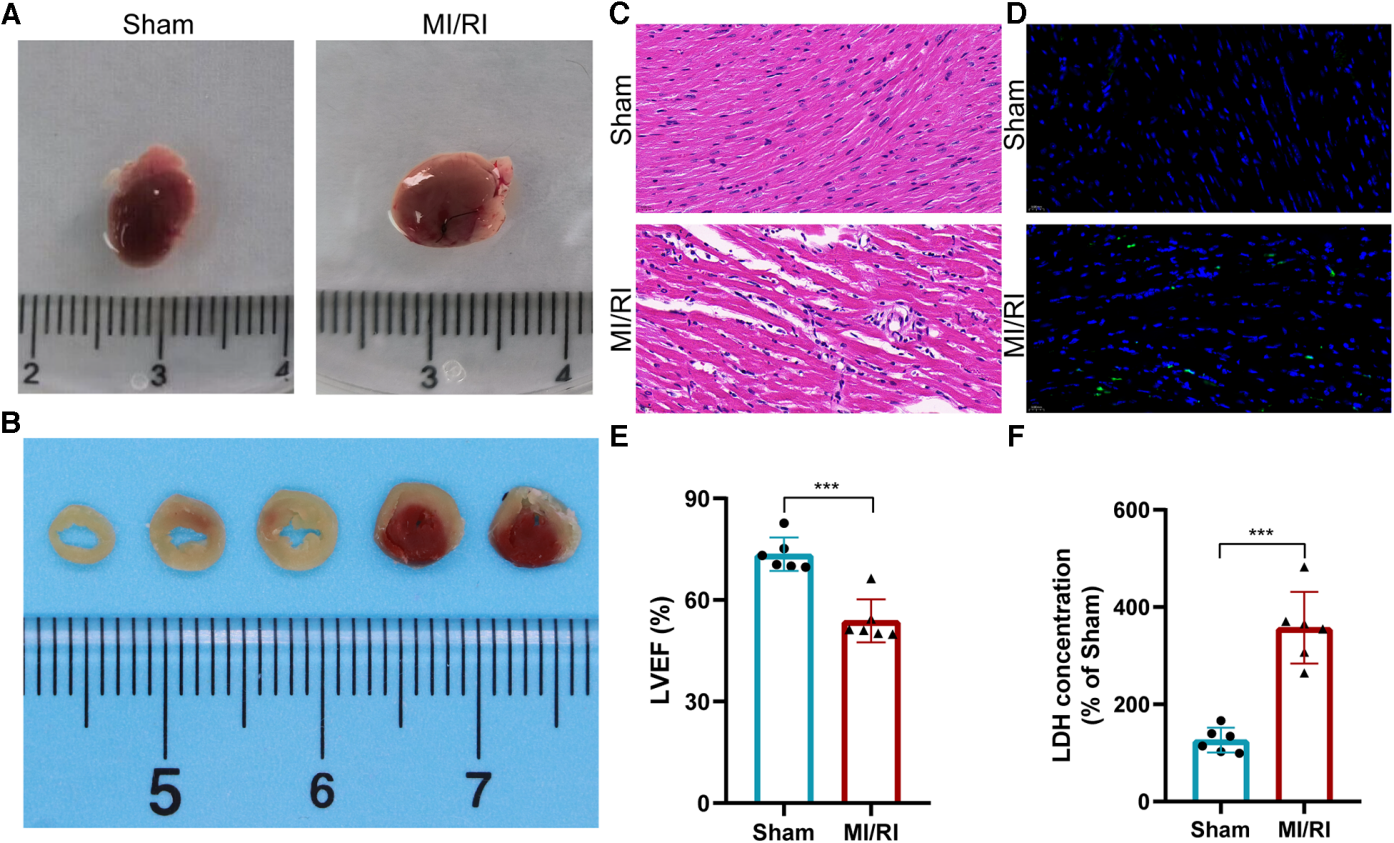
Establishment and evaluation of the mouse myocardial ischemia-reperfusion injury model. (**A**) Appearance of the heart. (**B**) TTC staining results of MI/RI model mouse heart. (**C–F**) HE staining (**C**), Tunel staining (**D**), LVEF detection (**E**), and serum LDH detection (**F**) results in the MI/RI model group and sham operation group. For panels (**E,F**), ***P < 0.001.

We used TTC staining, H&E staining, TUNEL staining, echocardiography, and serum LDH detection to verify the successful construction of the mouse MI/RI model. The results showed that despite the removal of polyethylene tube and restoration of blood supply, myocardial tissue in the MI/RI group of mice showed ischemic necrosis (Figures 9A,B). HE staining showed a disordered arrangement of cardiomyocytes and increased inflammatory cell infiltration after MI/RI treatment (Figure 9C). TUNEL staining showed that the apoptotic cells in the MI/RI group increased significantly (Figure 9D). In addition, LVEF was significantly decreased and serum LDH levels were significantly increased in the MI/RI group (Figures 9E,F). These results revealed the successful construction of the MI/RI model.

### Further validation of the identified key LMRGs in mouse myocardial ischemia-reperfusion injury model

**Figure 10:**
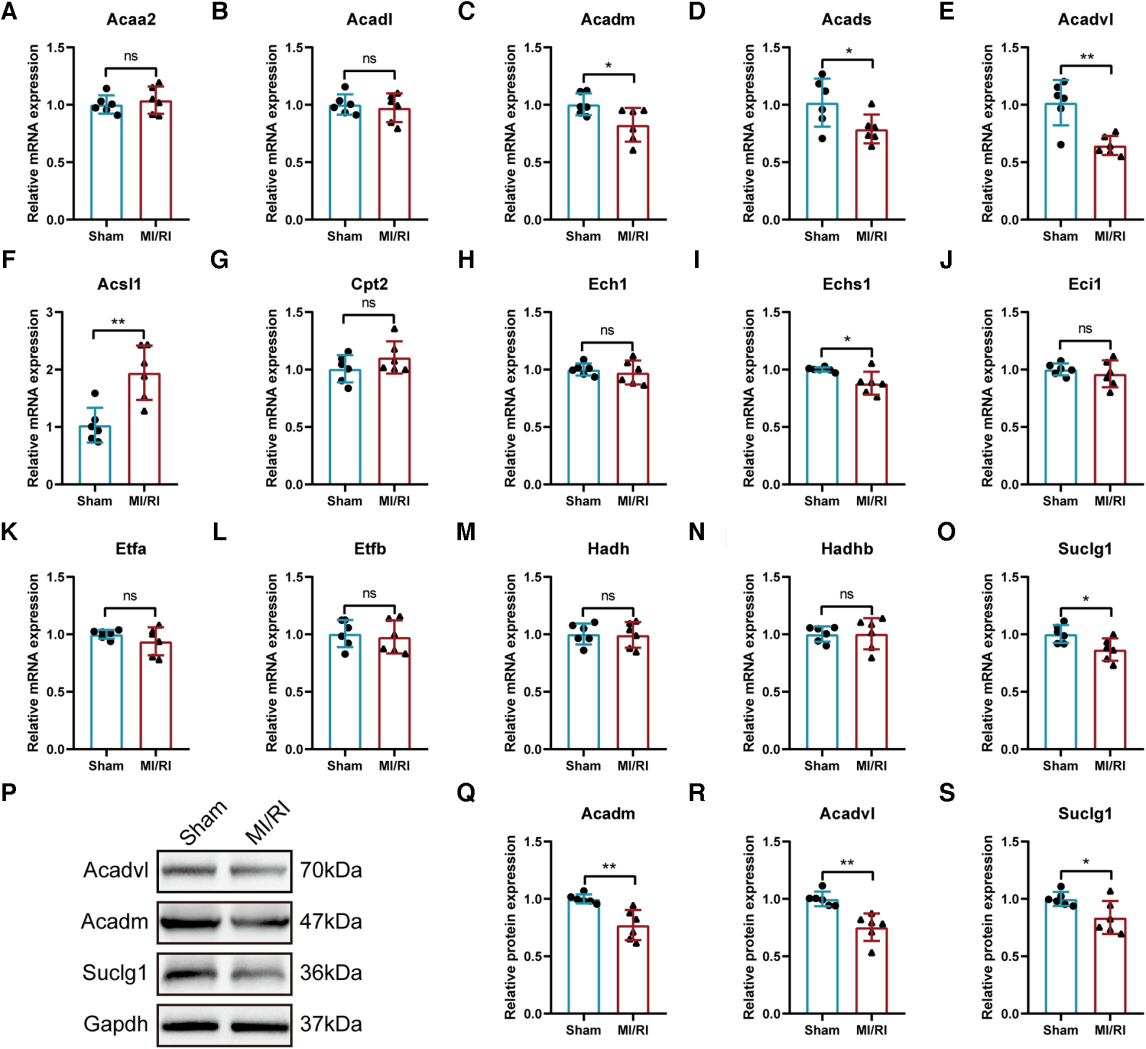
Further validation of the identified key LMRGs in mouse myocardial ischemia-reperfusion injury model. (**A–O**) Validation of 15 key LMRGs Acaa2, Acadl, Acadm, Acads, Acadvl, Acsl1, Cpt2, Ech1, Echs1, Eci1, Etfa, Etfb, Hadh, Hadhb, and Suclg1 in the mouse MI/RI model. *n* = 6 for each group. (**P–S**) Western blotting experiments detected the protein expression of Acadm, Acadvl, and Suclg1 in the mouse MI/RI model. For panels (**A–O, Q-S**), ns indicates no statistical difference; *P < 0.05, **P < 0.01.

The expression trends of the identified key LMRGs were further verified in the mouse MI/RI model (Figures 10A–O). The results showed that 6 LMRGs were differentially expressed in the mouse MI/RI model, among which *Acsl1* was significantly up-regulated, while *Acadm, Acads, Acadvl, Echs1, and Suclg1* were significantly down-regulated. The expression trends of 5 down-regulated genes (*Acadm, Acads, Acadvl, Echs1, and Suclg1*) were consistent with the prediction results; while up-regulated gene Acsl1 showed the opposite trend.

In conclusion, the expression trends of *Acadm, Acadvl, and Suclg1* were confirmed by the external datasets, the H/R model and the MI/RI model. Western blotting experiment found that the protein expression levels of these three genes were significantly down-regulated in MI/RI-treated mice (Figures 10P–S).

### Analysis of molecular docking results

**Figure 11:**
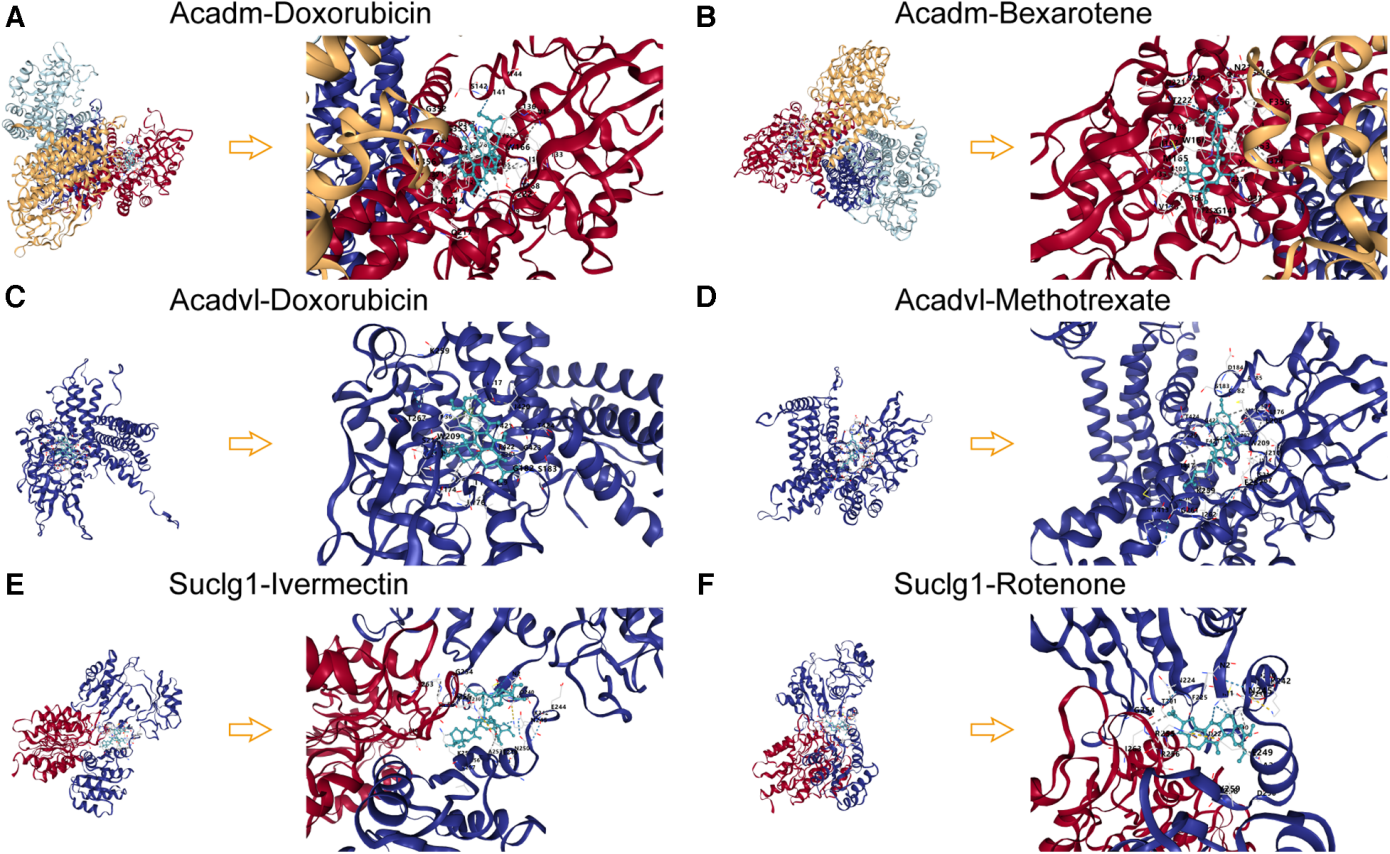
Molecular docking patterns of key LMRGs and therapeutic agents. (**A**) Molecular docking mode diagram of Acadm and Doxorubicin. (**B**) Molecular docking mode diagram of Acadm and Bexarotene. (**C**) Molecular docking mode diagram of Acadvl and Doxorubicin. (**D**) Molecular docking mode diagram of Acadvl and Methotrexate. (**E**) Molecular docking mode diagram of Suclg1 and Ivermectin. (**F**) Molecular docking mode diagram of Suclg1 and Rotenone.

Molecular docking of target agents and proteins expressed by key LMRGs was performed and Vina Score was calculated (Supplementary File S6). The results showed that Acadm and Doxorubicin (Vina Score = −11.7), Bexarotene (Vina Score = −11.3); Acadvl and Doxorubicin (Vina Score = −10.6), Methotrexate (Vina Score = −9.7); Suclg1 and Ivermectin (Vina Score = −9.4), Rotenone (Vina Score = −9.1) had lower Vina Sore and could bind stably (Figures 11A–F).

## Discussion

MI/RI is the main cause of poor revascularization outcomes after myocardial infarction. The process of ischemia-reperfusion affects the metabolic pathway of cardiomyocytes, causing the disturbance of energy balance and eventually leading to the injury of cardiomyocytes. Lipid metabolism is an important way of energy supply in cardiomyocytes; abnormal lipid metabolism can cause lipid accumulation in cardiomyocytes, mitochondrial dysfunction, and apoptosis (32). Therefore, the intervention based on lipid metabolism could be a new direction for the prevention or treatment of MI/RI.

This study used bioinformatics methods to perform data mining on the MI/RI-related transcriptome datasets. Differential expression analysis identified 855 DEGs in dataset GSE61592. Enrichment analysis suggested that lipid metabolism was the main biological process in which these DEGs were found to be involved. The obtained DEGs were intersected with LMRGs, and 120 differentially expressed LMRGs were obtained. The enrichment analysis results portrayed that the differentially expressed LMRGs identified were mainly involved in signaling pathways such as “fatty acid beta-oxidation”, “lipid transport”, “lipid storage”, “fatty acid biosynthetic process”, “PPAR signaling pathway”, “adipocytokine signaling pathway”, “peroxisome”, and “cholesterol metabolism”, etc. These identified pathways have been partially studied in regard to MI/RI. Progressive down-regulation of peroxisome proliferator-activated receptor γ (PPARγ) has been reported in patients with acute myocardial infarction undergoing coronary artery bypass grafting and in the mouse model of MI/RI. Activating the PPAR signaling pathway by melatonin could protect against MI/RI (33). Adiponectin is an adipocyte-derived plasma protein that has anti-diabetic and anti-inflammatory properties. Cardiac adiponectin induced by long-term insulin treatment can significantly reduce MI/RI in type 1 diabetic mice (34). These studies pointed out to the potential role of the identified signaling pathways for the treatment of MI/RI. Further studies are needed to explore their precise mechanism.

PPI analysis was performed on the 120 differentially expressed LMRGs, and 15 key LMRGs (*Acaa2, Acadl, Acadm, Acads, Acadvl, Acsl1, Cpt2, Ech1, Echs1, Eci1, Etfa, Etfb, Hadh, Hadhb, and Suclg1*) were identified. Notably, these key LMRGs were all down-regulated. GSE160516 contained mice myocardial expression profile data at 6 h, 24 h, and 72 h of reperfusion. Validation of the key LMRGs in GSE160516 showed that these genes were down-regulated except for Suclg1. Suclg1 increased significantly at 6 h of reperfusion and then decreased significantly at 24 h. GSE4105 contained rats' myocardial expression profile data at 2 days and 7 days of reperfusion. Validation of the key LMRGs in GSE4105 showed that these genes were all down-regulated. These validation results were consistent with the dataset analysis results.

The corresponding online databases predicted 126 miRNAs, 55 TFs, and 51 therapeutic agents targeting the key LMRGs. Among the predicted miRNAs, miR-5624-3p, miR-7092-5p, miR-6540-5p, miR-6911-5p, miR-7081-3p, miR-19b-3p, miR-19a-3p, miR-3090-3p, miR-205-3p target two or more LMRGs simultaneously. Among the predicted TFs, *Ppara, Rxrb, Rxra, Rxrg, Klf4, Cebpa, Stat5a, Med1, Smarcd3, Ncoa3, Ncoa6, Esrrb, Zfx, E2f1, Mycn, Pparg, Creb1* target two or more LMRGs simultaneously. These predicted molecules have been partially investigated in the setting of MI/RI. The miR-19 family has been reported to reduce MI/RI by directly inhibiting pro-apoptotic proteins (35). It has also been found that miR-205 can attenuate MI/RI by targeting ACSL4 to alleviate ferroptosis (36). Klf4 deficiency exacerbates MI/RI in mice by enhancing ROCK1/DRP1 pathway-dependent mitochondrial fission (37). Spherical *α*-helical polypeptide-mediated E2f1 silencing would also significantly alleviate MI/RI (38). In addition, 51 potential therapeutic agents such as estradiol, fenofibrate, resveratrol, sulforaphane, sunitinib, bezafibrate, propylthiouracil, pravastatin, quercetin, ciprofibrate were predicted to target the identified key LMRGs. Estrogen inhibits endoplasmic reticulum stress and reduces MI/RI by up-regulating SERCA2a in rats (39). Fenofibrate protects rats from MI/RI by inhibiting mitochondrial apoptosis (40). Resveratrol preconditioning can reduce MI/RI by regulating the AMPK pathway and autophagy level (41). Sulforaphane could protect from MI/RI through the balanced activation of Nrf2/AhR (42). The protective effect of quercetin on MI/RI has also been confirmed (43). These observations may suggest that the identified molecules and agents have significant potential value in the prevention or treatment of MI/RI. Further studies would be needed to elucidate their association with lipid metabolism and their mechanisms of action.

In addition, seven key LMRGs (*Acadl, Acadm, Acadvl, Etfa, Hadh, Hadhb, and Suclg1*) were verified in the H9C2 cell H/R model, which was consistent with the analysis results. *Acadl, Acadm, and Acadvl* are Acyl-CoA dehydrogenases that catalyze the oxidation of mitochondrial fatty acid β. A recent study highlighted the role of the KLF7/PFKL/ACADL axis in regulating myocardial metabolic remodeling during myocardial hypertrophy (44). Mice with Acadl or Acadvl defects also tended to display a phenotype of cardiac hypertrophy (45). *Etfa* transfers the electrons to the main mitochondrial respiratory chain via ETF-ubiquinone oxidoreducta (46). *Hadh* is a member of the 3-hydroxy acyl-CoA dehydrogenase gene family and catalyzes the oxidation of straight-chain 3-hydroxy acyl-CoAs. Hadh mutation causes mitochondrial dysfunction in neonatal rat hearts (47). *Hadhb* encodes the β-subunit of the mitochondrial trifunctional protein that catalyzes the last three steps of mitochondrial β-oxidation of long-chain fatty acids. Mutation of the Hadhb gene leads to a systemic disease with early onset cardiomyopathy i.e., fetal left ventricular non-dense cardiomyopathy (48). Suclg1 encodes the alpha subunit of the heterodimeric enzyme succinate coenzyme A ligase. Mutation of *Suclg1* causes mitochondrial DNA deletion and mitochondrial morphological changes (49, 50). These studies unveiled the role of the identified LMRGs in cardiomyopathy. Whether they would regulate lipid metabolism with a similar mechanism and affect MI/RI requires further investigation. In addition, the expression trend of some genes was not consistent with the prediction results, such as the up-regulation of genes *Acaa2, Acads, Echs1, and Eci1*, and no obvious trend of genes *Acsl1, Cpt2, Ech1, and Etfb*. These inconsistencies may result from the inconsistency between *in vitro* and *in vivo* experiments.

Finally, the down-regulation trend of *Acadm, Acads, Acadvl, Echs1, and Suclg1* was confirmed by *in vivo* I/R experiments. In summary, we found that *Acadm, Acadvl, and Suclg1* were significantly downregulated in MI/RI-related datasets GSE61592, GSE160516, and GSE4105 as well as in the *in vitro* H9C2 H/R model and in the *in vivo* MI/RI model. These genes may affect the pathophysiological process of MI/RI by regulating lipid metabolism in myocardial cells.

This study was based on transcriptome data mining, database prediction, and preliminary *in vitro* and *in vivo* verification and carried certain limitations. Further studies may be needed to explore the mechanism of the identified LMRGs and the therapeutic value of the identified agents. In addition, the identified drugs also need to be further tested in animal pharmacology and toxicology.

## Conclusions

In summary, this study used bioinformatics analysis methods to mine the MI/RI-related transcriptome datasets. Three key LMRGs (*Acadm, Acadvl, and Suclg1*) were identified, and 51 therapeutic agents (Such as estradiol, fenofibrate, resveratrol, sulforaphane, sunitinib, bezafibrate, propylthiouracil, pravastatin, quercetin, ciprofibrate) were predicted. These identified genes or molecules could provide new directions for the prevention or treatment of MI/RI.

## Data Availability

The original contributions presented in the study are included in the article/**Supplementary Material**, further inquiries can be directed to the corresponding authors.

## Supplementary material

Supplementary Material File S1 List of lipid metabolism-related genes.

Supplementary Material File S2 Establishment and evaluation of the mouse Myocardial Ischemia-Reperfusion Injury model.

Supplementary Material File S3 The primer sequence information of qPCR experiment.

Supplementary Material File S4 The detailed information of 855 DEGs.

Supplementary Material File S5 Venn diagram of key LMRGs predicted by multiple algorithms.

Supplementary Material File S6 Agent prediction and molecular docking results.
