# Supplementary material for: Transcriptomic analysis reveals the lipid metabolism-related gene regulatory characteristics and potential therapeutic agents for myocardial ischemia-reperfusion injury
Source: Front Cardiovasc Med. 2024 Jan 29;11:1281429. doi: 10.3389/fcvm.2024.1281429 (PMC10859419; doi:10.3389/fcvm.2024.1281429)
Supplement: Supplementary Material File S2 — Establishment and evaluation of the mouse Myocardial Ischemia-Reperfusion Injury model. [file Table2.docx]

***Construction of the mouse MI/RI model***

The mice were anesthetized by intraperitoneal injection of 1% pentobarbital sodium solution (0.60 ml/100 g). They were fixed on the operating table supine and underwent mechanical ventilation with oral tracheal intubation and electrocardiogram (ECG) monitoring. The chest was opened, the heart was exposed, and the LAD branch was lapped with 8.0 silk thread (a No. 10 polyethylene tube of 0.2 ~ 0.3 cm was placed under the line before ligation). The left ventricular anterior wall became white, local systolic motion was limited, and the left atrial appendage was full. The ECG showed continuous arched ST-segment elevation, which indicated successful myocardial ischemia. After 30 minutes of ischemia, the polyethylene tube was removed, the heart surface changed from pale to red, and the ECG showed partial resolution of ST-segment elevation, which indicated successful reperfusion. Finally, the chest was sutured and closed. After the mice recovered, the tracheal tube was removed and the mice were placed on a 38℃ thermal blanket overnight.

***TTC staining***

The heart tissues of mice were rinsed in pre-cooled PBS solution and refrigerated at -20 °C for 30 minutes. Then the tissues below the ligature were cut into 5 pieces on average along the longitudinal axis. The slices were placed in a 15 mL centrifuge tube containing 2% TTC dye and incubated at 37°C for 30 minutes away from light. Finally, the slices were immersed in 4% paraformaldehyde overnight.

***H&E staining***

Freshly isolated hearts were fixed with 4% paraformaldehyde, paraffin-embedded, and transversally sectioned (4-5μm). The heart sections were then stained with HE to evaluate the morphological characteristics of the heart. Stained heart tissue images were obtained by the Leica DMI3000B microscope.

***TUNEL staining***

Freshly isolated hearts were fixed with 4% paraformaldehyde, paraffin-embedded, and transversally sectioned (4-5μm). To assess apoptosis levels, sections of paraffin-embedded myocardial tissue were stained using the TUNEL Kit (Beyotime, Shanghai, China). Stained images were obtained by the inverted fluorescence microscope (Olympus, Tokyo, Japan), and the apoptotic cells were stained green.

***Echocardiographic evaluation***

Left ventricular ejection fraction (LVEF) was measured by M-mode echocardiography to evaluate cardiac function.

***LDH assay***

The relative content of serum LDH in mice was measured by the LDH assay kit (Beyotime, Shanghai, China). LDH levels were measured at 450 nm using the automated porous spectrophotometer (Bio-Rad Laboratories, USA).
