# Supplementary figures and images for: Transcriptomic analysis reveals the lipid metabolism-related gene regulatory characteristics and potential therapeutic agents for myocardial ischemia-reperfusion injury
Source: Front Cardiovasc Med. 2024 Jan 29;11:1281429. doi: 10.3389/fcvm.2024.1281429 (PMC10859419; doi:10.3389/fcvm.2024.1281429)

**Venn diagram of key LMRGs predicted by multiple algorithms**


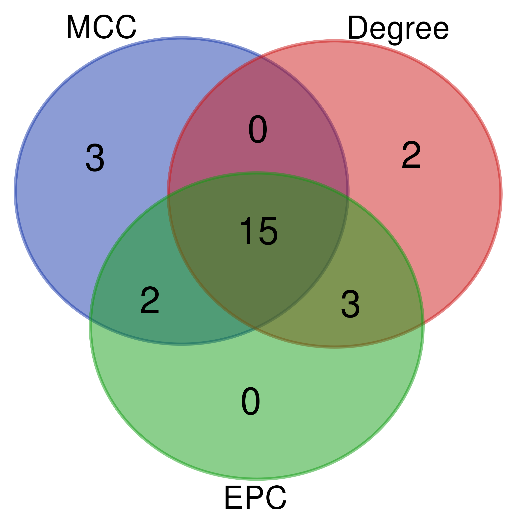

Supplement: Supplementary Material File S5 — Venn diagram of key LMRGs predicted by multiple algorithms. [file Table5.docx]
